# Co-Occurrence of Tic Disorders and Attention-Deficit/Hyperactivity Disorder—Does It Reflect a Common Neurobiological Background?

**DOI:** 10.3390/biomedicines10112950

**Published:** 2022-11-17

**Authors:** Aribert Rothenberger, Hartmut Heinrich

**Affiliations:** 1 Clinic for Child and Adolescent Psychiatry and Psychotherapy, University Medical Center Göttingen, 37075 Göttingen, Germany; 2 Neurocare Group, 80331 Munich, Germany; 3 Kbo-Heckscher-Klinikum, 81539 Munich, Germany; 4 Research Institute Brainclinics, Brainclinics Foundation, 6524 AD Nijmegen, The Netherlands

**Keywords:** tic disorders, attention-deficit/hyperactivity disorder, comorbidity, genetics, neuroanatomy, neurochemistry, neurophysiology, additive model

## Abstract

Background: The co-existence of tic disorders and attention-deficit/hyperactivity disorder (TD + ADHD) has proven to be highly important in daily clinical practice. The factor ADHD is not only associated with further comorbidities, but also has a long-term negative psychosocial effect, while the factor TD is usually less disturbing for the major part of the patients. It remains unclear how far this is related to a different neurobiological background of the associated disorders or whether TD + ADHD reflects a common one. Objective: This review provides an update on the neurobiological background of TD + ADHD in order to better understand and treat this clinical problem, while clarifying whether an additive model of TD + ADHD holds true and should be used as a basis for further clinical recommendations. Method: A comprehensive research of the literature was conducted and analyzed, including existing clinical guidelines for both TD and ADHD. Besides genetical and environmental risk factors, brain structure and functions, neurophysiological processes and neurotransmitter systems were reviewed. Results: Only a limited number of empirical studies on the neurobiological background of TD and ADHD have taken the peculiarity of co-existing TD + ADHD into consideration, and even less studies have used a 2 × 2 factorial design in order to disentangle the impact/effects of the factors of TD versus those of ADHD. Nevertheless, the assumption that TD + ADHD can best be seen as an additive model at all levels of investigation was strengthened, although some overlap of more general, disorder non-specific aspects seem to exist. Conclusion: Beyond stress-related transdiagnostic aspects, separate specific disturbances in certain neuronal circuits may lead to disorder-related symptoms inducing TD + ADHD in an additive way. Hence, within a classificatory categorical framework, the dimensional aspects of multilevel diagnostic-profiling seem to be a helpful precondition for personalized decisions on counselling and disorder-specific treatment in TD + ADHD.

## 1. Introduction

More than a decade ago, a group of experts presented a collection of articles with an overview concerning different aspects (including epidemiology, neurobiological background, treatment) of the clinically important co-existence of tic disorders and attention-deficit/hyperactivity disorder (TD + ADHD) [1] (see also Box 1). For example, in a large cross-sectional international clinical cohort study of 6805 individuals with TD, 61% of children and 39% of adults were diagnosed with TD + ADHD, where the factor ADHD was strongly associated with further comorbidities and psychosocial problems [2].

In the meantime, large-scale studies shed further light on the prevalence and impact of TD + ADHD. Scharf et al. [3] reported data of 13-year-olds from the Avon longitudinal study cohort (*n* = 6768), indicating that co-occurring ADHD in TD cases is markedly lower in population-based samples (compared to clinical samples).

The negative psychosocial impact of TD plus co-occurring conditions was outlined by Bitsko et al. [4] among a nationally representative sample of US children (*n* = 65,540, aged 6–17 years). After controlling for co-occurring neurobehavioral conditions (e.g., school problems, unmet mental health care needs) the before negative findings were no longer significant for the TD group, Ricketts et al. [5] studied three groups, namely TD, ADHD, and TD + ADHD (*n* = 3014, age 4–17 years) and found that children with TD + ADHD had higher mean impairment across all domains (e.g., problematic handwriting and interpersonal relationships) than children with either TD or ADHD. The authors concluded that disorder-specific contributions to impairment could inform targeted interventions for TD and ADHD within TD + ADHD.

Box 1 **Tic disorders, ADHD and comorbidity at the clinical level.**

The most comprehensive and updated textbook on Tourette Syndrome with a worldwide group of experts [6] includes clinical phenomenology (also with comorbidities), epidemiology with prevalence, etiology, pathophysiology, diagnosis, assessment, treatment, resources, and support. Here, only necessary information concerning TD + ADHD is given.

Chronic tic disorders (prevalence of about 1–4% depending on subtype) are basically characterized by waxing and waning motor/vocal tics, with an onset around the age of seven years, increasing in frequency, diversity, and severity up to the age of 12–14 years and declining thereafter. Around the age of 10 years, additional premonitory phenomena (e.g., sensorimotor premonitory urges to tic) may be recognized by the child. Tics can be suppressed for a certain amount of time. This natural compensatory neuronal process using prefrontal mechanisms [7] can be integrated into more sophisticated cognitive-behavioral treatment approaches, either with/without dopamine blocking agents according to existing guidelines (see [8,9,10,11]).

ADHD (prevalence of about 3–6%) is also a neurodevelopmental disorder that presents with symptoms of inattention and/or hyperactivity–impulsivity. According to DSM-5, three subtypes/presentations are defined, namely inattentive, hyperactive/impulsive, and combined. ADHD can be diagnosed from the age of four years onward, often persists into adulthood, and is usually accompanied by psychosocial impairment. Treatment with behavioral therapy and/or stimulants/noradrenergic drugs is recommended according to existing evidence-based conclusions [12].

In both TD and ADHD, about 70–80% of individuals show at least one additional behavioral problem. In TD, the co-occurrence of ADHD can be found in about 50% of patients (in clinical samples), while in ADHD about 20% of cases also present with TD. Both percentages are well above what would be expected by chance alone [13]. Besides ADHD, obsessive-compulsive disorder (OCD) is another important comorbidity with TD (TD in OCD of about 30%, OCD in TD 10–35%; [14]).

Comorbidity of TD in ADHD has low or no impact on impaired psychosocial functioning and learning, while comorbidity of ADHD in TD is highly associated with additional cognitive and psychosocial problems as well as with the appearance of further psychopathologies [1,13,15]. This underlines the clinical and even subclinical importance of this issue and demands further research in order to clarify the neurobiological background, thus providing a sound basis for better diagnostics and treatment.

According to the variety of the developmentally determined core symptoms, their spectrum of severity and associated psychopathologies TD, ADHD, and TD + ADHD may present with a great heterogeneity. This makes it highly challenging for diagnostics, treatment, and research.

The long-term negative effect of ADHD can be found even beyond psychosocial aspects. This was underlined recently by Brander et al. [16]. They investigated in a large longitudinal population-based cohort study in Sweden the risk of metabolic and cardiovascular disorders among individuals with TD over a period of 40 years, which was increased. The latter was particularly the case in those with comorbid ADHD, assuming that ADHD might just add its own risk when it comes to TD + ADHD comorbidity, or ADHD might be the driving interactive force in developing associated somatic and/or neuropsychiatric problems in TD + ADHD.

It remains unclear how far this is related to a different or a common neurobiological background of the two associated disorders in TD + ADHD.

This review will provide an update on the neurobiological background of TD + ADHD, having in mind that growing new empirical evidence within this area of research is needed to give rise to a better understanding and treatment of this clinical problem. Within this context, it should be investigated whether a specific role of each factor (i.e., TD vs. ADHD) can be stated or not. In addition, it should be discussed whether, within such a classificatory framework, the dimensional aspects of transdiagnostic neurobiological overlap may play a role. Specifically, in TD and ADHD, categorical as well as dimensional approaches are worth following in clinical practice and research: having or not having a tic is clearly categorical, while attention, motor activity, and impulsivity are dimensional. This review is phenotypically guided by the clinical categories as defined in DSM-5 while looking from this standpoint to a specific/non-specific neurobiological background.

**Figure 1:**
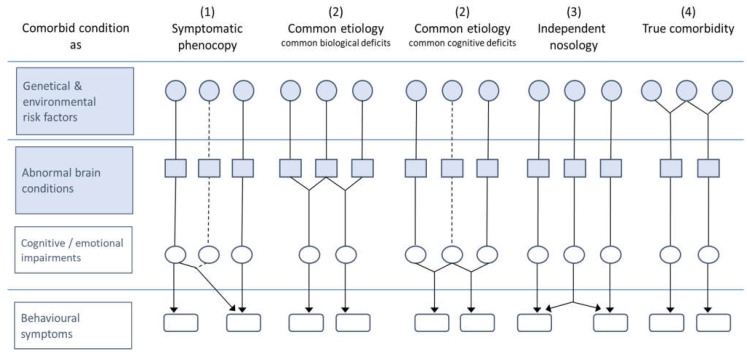
Models of comorbidity (adapted from Banaschewski et al. [15]; with permission of Springer). In the present article, only levels “Genetical/environmental risk factors” and “Abnormal brain conditions” are considered.

When modelling the co-occurrence of TD + ADHD, it has to be clarified whether this combination represents two independent pathologies (reflecting an additive model), a separate nosological entity manifested by both tics and ADHD symptoms (interactive model), or a phenotypical subgroup of one of the two major clinical forms (phenotype model), as outlined in Banaschewski et al. [17] and Rothenberger et al. [1] (see also Figure 1).

So far, a stepwise model for children and adolescents has been proposed, i.e., the co-occurrence of TD + ADHD might follow an additive model at the neurobiological level (e.g., sleep) while with higher cognitive functions/performance (e.g., executive functions) a merely interactive modelling might come into play (e.g., it remains open whether this really holds for increased event-related theta activity in TD + ADHD [18]). Because data for adults are scarce, the respective modelling still has to be done (e.g., an additive model for psychopathological symptoms is questioned in young adults [19]). At the moment, in children and adolescents the ADHD part of TD + ADHD should be the primary goal for treatment, while in adults both disorders may be handled equally. Hence, this review will investigate whether the empirically-based assumption of an additive model for TD + ADHD still holds true or whether new empirical research demands another kind of modelling for TD + ADHD with different clinical recommendations.

To answer this question methodologically, a 2 × 2 factorial design of studies would be best to disentangle the factors TD vs. ADHD, i.e., optimally, studies should include four groups of patients, namely TD-only, ADHD-only, TD + ADHD, and typically developing probands (TDP). Concerning clinically oriented examples see Roessner et al. [20] for psychopathology and Greimel et al. [21] for neuropsychology. Because no interactions between both factors were found, in these cases an additive model was assumed. This suggests (at these levels) that both disorders should be seen as separate entities to be treated in parallel, depending on their psychosocial impact.

The assumption of behavioral additivity for TD and ADHD is also supported by a longitudinal study on the clinical course of TD. In a cohort of, at baseline *n* = 314 patients with TD (ages 5–19 years), and at follow-up 6 years later (*n* = 227, ages 11–26 years), changes in the expression of tics and comorbidities were examined. There was found a significant decline of both TD and ADHD, while there were still 63% of co-existing psychopathologies at follow-up (not only TD + ADHD). The strongest predictors for future symptoms of TD or ADHD were the respective diagnoses at baseline. This stability of symptoms over time in parallel may be a hint for separate lines of development for TD and ADHD, even within TD + ADHD [22].

Deriving from behavioral findings and earlier neurobiological reports in TD + ADHD [17], with their empirical evidence for the additive model of TD + ADHD and its use in clinical practice, this review investigates whether this model still holds, is strengthened, or even has to be refused while considering more recent data. Although many studies on the neurobiology of TD + ADHD did not use the favorable 2 × 2 design, they may be helpful for clarification of the mechanisms behind this co-occurring picture and thus will also be included in this narrative review, which always has a clinical perspective in mind. The review is based on a literature research in PubMed (updated on 1 September 2022; keywords: tic disorder/Tourette; ADHD; comorbidity and related terms); the references of the papers found were also screened and clinical guidelines for TD [23,24] and ADHD [12,25] were used. 

## 2. Level “Genetical & Environmental Risk Factors”

### 2.1. Familiality and Genetics

As a precondition of a common genetic background, TD + ADHD should run in families. Earlier studies with small sample sizes suggested that there may exist an increased risk of ADHD in relatives of TD probands and also an increased risk of TD + ADHD in families affected with this comorbidity [26,27]. In addition, Mathews and Grados [28] stated that familiality/heritability between TD and ADHD may be due to a genetic association between OCD and ADHD. This may mean that subcategorical ADHD (and OCD) symptoms in TD may play a role in this respect. To better clarify this issue, Roessner et al. [29] analyzed a large sample of ADHD-affected children and their (partly also affected) siblings. The study was based on the International Multicenter ADHD Genetics (IMAGE) project. The investigated sub-samples of 2815 individuals, about 11 years old, included ADHD-index patients with co-existing TD (ADHD + TD, *n* = 262) and without TD (ADHD-TD, *n* = 947) as well as their 1606 full siblings (*n* = 388 of the ADHD + TD index patients and *n* = 1248 of the ADHD-TD index patients). It was tested whether the risk for ADHD, TD, and ADHD + TD in siblings was associated with the related index patients’ diagnosis. In order to obtain an estimate for psychopathological specificity, the four groups were also compared for general psychopathological symptoms using the strengths and difficulties questionnaire (SDQ).

The main finding was a significantly higher risk to present with ADHD + TD (and a tendency for TD without ADHD) among siblings of patients with ADHD + TD. This suggests that the familiality of ADHD + TD may exist, probably driven by the factor TD, which is part of both groups, namely ADHD + TD and TD without ADHD. There was no significant hint for a higher general psychopathological vulnerability in siblings of index patients with ADHD-TD, and thus the results may reflect some psychopathological specificity for TD as well as an additive model for TD + ADHD.

If familiality of TD + ADHD is true, the question remains, what kind of genes are responsible for the genetic background?

Both TD and ADHD seem to be heterogeneous polygenic disorders (e.g., see review for genetics in TD and its relationship with other psychiatric disorders by Qi et al. [30]) due to the small but additive effect of multiple genes of a different kind, e.g., genes affecting dopamine, norepinephrine, serotonin, GABA, and other neurotransmitters (although known as principally transdiagnostic) may finally make up a more or less disorder-specific genetic and thus also dimensional neurotransmitter profile, which may help to differentiate “categorical neurobiological entities” behind a certain clinical picture, providing a basis for the testing of an additive model of TD + ADHD in a 2 × 2 statistical design. Although psychiatric disorders (with their mostly overlapping dimensional symptoms such as attentional problems) share common variant genetic risks, neurological disorders (such as tics in TD) appear more distinct from one another and from psychiatric disorders [31]. There exists some overlap of genes between TD and ADHD. For example, Gomez et al. [32] reported a risk gene for both TD and ADHD in the *11q24* chromosomal region. Tested in *n* = 174 nuclear families with TD and *n* = 242 nuclear families with ADHD, the study indicated that the potassium inwardly-rectifying channel J5 (*KCNJ5*) was associated with TD and with ADHD, but functional variants remain to be identified and a TD + ADHD group was not investigated.

Further, Janik et al. [33] compared *n* = 162 patients with TD with *n* = 180 healthy probands according to the *BTBD9* gene polymorphisms and came to the conclusion that these gene variants are not associated with TD but with ADHD comorbidity. Unfortunately, an ADHD-only group was not also investigated.

Tsetsos et al. [34] investigated, for the first time, the common genetic background of TD and ADHD, applying Genome Wide Association Studies (GWAS) in large samples with TD and ADHD, respectively. Their main finding for both disorders refers to the Ras signaling cascade as cross-disorder key elements of the brain signaling pathways. Recently, Yang et al. [35] investigated the genetics of TD, ADHD, ASD (autism spectrum disorder), and OCD (obsessive compulsive disorder) as disorders with the possibility of a common cross-disorder genetic architecture along a dimensional impulsivity–compulsivity spectrum. They applied integrating summary statistics from the latest genome-wide association studies (93,294 individuals; 6,788,510 markers) and uncovered 13 genome-wide significant regions, probably associated with three of the studied disorders, namely TD, ADHD, and ASD. Cross-disorder tissue specificity analysis implicated the hypothalamus-pituitary-adrenal gland-axis and might imply that some weakness of the general stress system is the unifying biological factor related to this shared genetic basis. From clinical experience, the influence of stress on tics can be either decreasing or worsening. Probably because of this heterogeneity, Buse et al. (2022) could not find a link between hair cortisol concentration as a stress marker and the severity of perceived psychosocial stress in a European cohort (COURSE sample) of *n* = 412 children and adolescents aged 3–16 years with a chronic tic disorder (CTD) including TS and *n* = 131, 3–10 years old non-tic siblings of CTD individuals. Further, cross-disorder non-specific neurobiological dysfunctions exist (e.g., see [36] for TD: ion channel signaling, cell adhesion, transsynaptic signaling processes), which can be considered as dimensional cross-disorder elements. For these, a common genetic basis may be found for TD and ADHD.

Another aspect of cross-disorder genetics between TD and ADHD, namely their common characteristic being an early-onset neurodevelopmental disorder, was outlined in a mega-sample paper of the Cross-Disorder Group of Psychiatric Genomics Consortium [37] and the review on pleiotropy by Lee et al. [38], but no comorbid groups were investigated explicitly. Hence, the genetic relationships between TD and ADHD seem to concern more general, non-specific/transdiagnostic aspects of development and/or subclinical ADHD symptoms within a TD diagnosis.

However, despite some general genetic overlap, both TD and ADHD must have different, specific genetic profiles/loadings related, for example, to hyper-dopaminergic activity in TD and hypo-dopaminergic function in ADHD. Both correspond to treatment with dopamine blocking agents such as tiapride and aripiprazole for TD and stimulants such as methylphenidate and noradrenergics such as atomoxetine for ADHD [24].

Because noradrenergic drugs such as guanfacine/clonidine/atomoxetine may be helpful for ADHD as well as TD, these substances may be good candidates to treat TD + ADHD. Hence, noradrenergic genes might reflect neurobiological commonalities and thus elucidate the neurobiological background of TD + ADHD. Xu et al. [39] investigated the potential role of adrenergic *ADRA2A* genetic variants. Two single nucleotide polymorphisms (SNPs) of *ADRA2A* were genotyped and analyzed in 936 normal controls and 1815 ADHD probands, including 1249 trios. Approximately 16% (*n* = 290) of the ADHD probands had TD + ADHD. While no significant association was found between *ADRA2A* and ADHD, the allelic/genotypic distribution and allelic transmission were different between TD + ADHD vs. TD-ADHD (i.e., TD without ADHD), especially in males. Unfortunately, there was no group with TD-only, and thus it can only be speculated that the *ADRA2A* gene effect might be related to the TD part of TD + ADHD, supporting an additive model of this comorbidity.

Probably, the genetics of TD + ADHD reflects two different neuronal mechanisms: one related to “non-additive” cross-disorder genes influencing more joint functions such as stress regulation, and another “additive” one that creates disorder-specific disturbances in certain neuronal circuits, leading to specific clinical symptoms.

### 2.2. Environmental Factors

As in other neurodevelopmental disorders, pre- and perinatal complications are known in TD and ADHD. However, what the role is of pre- and perinatal factors in relation to the expression of single diseases and moreover a comorbidity such as TD + ADHD remains an unresolved issue. In a literature review and critical commentary on epidemiological works evaluating the association between pregnancy-related and birth-related adversities, Hoekstra et al. [40] concluded that maternal smoking during pregnancy and low birthweight are risk factors for the presence of comorbid ADHD in individuals with TD. Further, on the basis of data from a large sample of patients with TD, Abdulkadir et al. [41] suggested that, in TD, adverse situations at birth or during the first weeks of life are largely associated with co-occurring ADHD. This is underlined by Brander et al. [42], who conducted a Swedish total population sibling comparison study and found a dose-response relationship between the number of adverse perinatal events and increased risk for TD, but the association with maternal smoking during pregnancy was no longer significant after the exclusion of comorbid ADHD. Hence, early environmental risk factors are more associated with the factor ADHD, which also seems to be (as tic severity) associated with clinically relevant explosive outbursts [43]. A summary of the findings is provided in Table 1.

## 3. Level “Abnormal Brain Conditions”

### 3.1. Brain Structure

Basal ganglia (BG) dysfunction is known from several developmental disorders, but the development of its functional connectivity with other brain regions over time is not well understood. Greene et al. [59] investigated *n* = 120 healthy adults (19–31 years old) and *n* = 60 healthy children (7–12 years old) by resting-state functional connectivity MRI (rs-fc MRI) and found a decrease in functional connectivity of BG with the cortical somatomotor face system, expressed by a partial correlation coefficient. The authors speculated (referring to facial tics) that the developmental trajectory of BG functional organization might be atypical in TS and that a refined rs-fc MRI coefficient might be used for diagnostic purposes.

Localizing neuroanatomical deviations of the brain has increased our understanding of psychiatric disorders within the last decade, specifically when developmental aspects could be detected [48]. However, we are far from diagnosing single disorders by their neuromorphological features, although some researchers could demonstrate initial proof-of-concept studies in participants with well characterized psychiatric illnesses, including TD and ADHD [60,61]. Bansal et al. state that their algorithmic approach “is yet unsuited for discrimination as a complete and practical tool to aid in clinical diagnosis” [60] (p. 19). It is more difficult to find a solution for morphometrically diagnosing comorbid disorders such as TD + ADHD. Nevertheless, there exist some hints as to which parts of the brain may be involved and how these might be different from TD-only and ADHD-only, respectively.

Plessen et al. [45] summarized the findings from structural neuroimaging studies of TD + ADHD. For the *basal ganglia*, they concluded that in TD there exist smaller caudate nuclei in both children and adults, which may suggest that the hypoplasia of the caudate nucleus represents a trait morphological abnormality in persons with TD, while the co-occurrence of ADHD did not statistically alter these findings.

Concerning *cortical regions*, they detected a higher proportion of white matter in the right frontal lobe of 11 boys with TD, but not in 14 boys with TD + ADHD, 12 with ADHD-only, or 26 healthy controls. Both individuals with TD + ADHD and ADHD-only had smaller overall frontal lobes, which might point to a driving effect of ADHD in these cases. However, one could not statistically distinguish morphometric differences of the brain that were specific to either TD or ADHD or TD + ADHD.

Finally, the *corpus callosum* (CC) was considered because of earlier findings of reduced brain asymmetry in the basal ganglia of TD patients, which has not been confirmed so far. However, DTI (Diffusion Tensor Imaging) findings seem to point to a reduced interhemispheric connectivity in children with TD. A comorbid diagnosis of ADHD did not influence the results [45].

In conclusion, the authors suggest that comorbid ADHD does not significantly alter the primary findings derived from samples with TD patients, thus providing only a little support of a shared genetic vulnerability as expressed in deviations of brain morphology in TD + ADHD.

Both TD and ADHD have been associated mainly with the structural variation of frontal lobes and striatum/basal ganglia. Taking this in focus, Forde et al. [62] looked at the basal ganglia structure in TD and/or ADHD by applying T1-weighted fMRI in *n* = 141 children between 8–12 years old. The analysis of basal ganglia volume or shape did not find any evidence that TD, ADHD, or TD + ADHD are associated with structural variations compared to healthy controls. There was also no association with ADHD severity and no interaction between TD and ADHD—the latter is also statistically essential for the assumption of an additive model in TD + ADHD.

Within the last decade, preliminary evidence was presented that the *limbic system* (“emotional brain”, see Pessoa and Hof [63]) may also be altered in both TD and ADHD [46,47], because emotional aspects and stress reactivity cannot be ignored in these patients, where the factor ADHD is probably responsible for weakness in emotional regulation [64]. This shows that emotion and cognition are highly integrated in a bidirectional fashion, e.g., the hypothalamus and the amygdala are connected throughout the frontal cortex. To fully understand the “emotional brain”, it is necessary to also understand the “cognitive brain”, and vice versa [63].

The small study of Ludolph et al. [46] aimed to investigate in vivo possible morphological alterations of the *amygdala* as a key component of the limbic system. Amygdala and total brain volumes were measured with MRI in 17 male patients with TD, including eight with TD + ADHD (mean age 11; 8 years) and 17 age-matched healthy controls (mean age 12; 6 years). There was an amygdala volume reduction in TD that did not correlate with tic severity but with symptoms of ADHD (and its accompanying behavioral impairment). Hence, primarily ADHD might be related to vulnerability of the amygdala, giving input of neuronal dysregulation to the striatum and frontal cortex. The latter usually may be able to compensate this disturbance, at least partly, and may be trained by neurofeedback in order to better regulate behavior [65].

A similar approach was reported in adults (mean age 30; 7 years) by Wittforth et al. [47]. A group (*n* = 12) including patients with TD + OCD + ADHD (*n* = 8) and TD + ADHD (*n* = 4) was compared to *n* = 17 with TD + OCD and *n* = 24 healthy controls by voxel-based morphometry (VBM). They found decreased grey matter volume in the left inferior frontal gyrus (IFG). By comparing TD + ADHD with TD without ADHD they registered a left sided amygdala volume increase. Although this study had only a very small subgroup of four patients with TD + ADHD, the deviation of amygdala volume for TD + ADHD in both studies (children and adults) suggests the importance of investigating the role of emotional regulation related to this brain area in TD + ADHD further.

Within this context, it seems worthwhile to have a critical eye on some results on brain volume/cortical thickness that assume morphometric deviations in TD extend from the motor cortex to the limbic system depending on the severity/complexity of tics and comorbidities [66]. For example, Draganski et al. [48] reported MRI data of 40 adults with TD (including *n* = 11 with TD + ADHD). The multiple morphometric changes, i.e., grey matter volume, decrease of hippocampus, putamen, and parietal cortex, were modulated by the presence of ADHD assuming an additive effect.

### 3.2. Neurochemistry/Neurotransmitters

Dopaminergic abnormalities in the striatum, although of a different kind, seem to play a key role in the pathophysiology of both TD and ADHD [8,12]. While TD is related to a hyper-dopaminergic mechanism, ADHD is merely a hypo-dopaminergic disorder. Because these striatal dysfunctions are located topographically close to each other, a high rate of TD + ADHD co-occurrence may not be surprising; all the more, the integrative interplay with several other fronto-striatal circuits has to be taken into account. Hence, the influence of serotoninergic, cholinergic, noradrenergic, glutamatergic, GABAergic, and endocannabinoid systems on TD and ADHD should also be investigated, because it seems plausible that, at least in TD + ADHD, multiple neurotransmitter systems may be involved.

The dopaminergic neurotransmission and its metabolism in the central nervous system of non-medicated patients with TD (*n* = 7), TD + ADHD (*n* = 12), and healthy controls (*n* = 19) was recently investigated by Capetian et al. [49]. They determined Tetrahydroisoquinolines (TIQs; modulating dopamine and thus giving another perspective on monoaminergic dysfunction [67]) in the urine by a two-phase chromatographic approach. TIQs were significantly highest in TD + ADHD, suggesting a dopaminergic hyperactivity for this comorbid psychopathology and underlining the role of dopamine in this case. Unfortunately, a group with ADHD-only was not included. Thus, it remains unclear which factor (i.e., TD vs. ADHD) was mainly responsible for this result and whether it can be explained by additivity.

Nevertheless, the overwhelming influence of a hyper-dopaminergic state in TD was also documented by Gilbert et al. [50], who could show in a four-week randomized, double-blind, placebo-controlled, crossover study in 40 youngsters with TD (aged 7–17 years) that even a D1-receptor antagonist (here: Ecopipam) can be well tolerated and can reduce tics with no changes in ADHD symptoms. Whether this may be seen as an extension of the well-based treatment of TD with D2-receptor blockers has to be investigated further. At least, these results do not contradict the drug-guided additive-model-hypothesis in TD + ADHD.

Besides dopamine, noradrenaline seems to be an important player in TD + ADHD, as can be seen from a clinical trial in *n* = 136 children with TD + ADHD. Either clonidine (CLON, a noradrenergic agonist) or methylphenidate (MPH, a dopaminergic agonist) or either their combination, CLON + MPH, or placebo were administered in a 2 × 2 factorial design over 16 weeks. CLON + MPH best reduced tics and ADHD symptoms, suggesting an additive effect of both neurotransmitters in TD + ADHD [68]. The differential effect of treatments on ADHD and tic symptoms suggests different (perhaps independent) neurobiological bases.

Both TD and ADHD have also been related to abnormalities in glutamatergic neurochemistry in the fronto-striatal circuitry, because of the importance of glutamate in the excitatory aspects of neurons, assuming higher concentrations of glutamate at least in the striatum of TD patients [69]. To further explore this issue, the authors used proton magnetic resonance spectroscopy (1H-MRS) in children between 8 and 12 years of age in a 2 × 2 factorial design (TD *n* = 15, ADHD *n* = 39, TD + ADHD *n* = 29, and healthy controls *n* = 53) to address confounds of comorbidity. Glutamate concentrations were evaluated in the anterior cingulate cortex (ACC) and the left dorsal striatum (LDS). Unexpectedly, no group differences could be found. Furthermore, variation in glutamate concentration in ACC and LDS did not show any relation to age, sex, medication use, IQ, tic, or ADHD severity. Obviously, there exists no empirical evidence for glutamatergic neuropathology in TD and/or ADHD.

Within the last decade some small clinical studies in adults and single-case observations in adults and adolescents suggest that cannabis-based-medicine (CBM) such as delta-9-tetrahydrocannabinol (THC), dronabinol, or street-cannabis may improve tics, and thus the endocannabinoid system (ECS) might be involved in the pathophysiology of TD [51]. So far, it is unclear whether the alterations of ECS are related to primary neuronal disturbances in TD, represent a secondary change within a compensatory mechanism for other more basal neurochemical deviations in the same was as dopamine, or whether it reflects just an epiphenomenon. The relationship to TD + ADHD comorbidity remains an open question, although in a chart-review of *n* = 38 adult patients with TD, these reported in 55% an improvement of comorbidities, including ADHD symptoms [70], suggesting an effect on disorder non-specific neuronal systems.

GABAergic alterations in different functional domains of the striatum might help to elucidate the co-occurrence of TD + ADHD. Examining the neuronal activity of freely behaving rats before and after domain-focused injections for neuronal disinhibition, Israelashvili et al. [71] found that injections within the striatal motor domain elicited merely stereotypic movements, while injections into the limbic domain induced mainly locomotor hyperactivity. This was interpreted as distinct tic-like and ADHD-like symptom expression, respectively, and could go well with an additive model for TD + ADHD.

Further, indicators for a higher immune activation in TD may exist (e.g., elevation of neopterin [72]), but it has still to be proven if streptococcal infections and ADHD are the driving force, and what this might mean for TD + ADHD.

In a recent review on immunology in TD and ADHD, Martino et al. [73] stated that (1) TD and ADHD have been associated with increased risk for autoimmune disorders and allergic conditions and (2) the potential correlation between infections (e.g., postnatal Group A Streptococcus, GAS [74]) and the development/exacerbation of TD remains questionable. This lack of an association was supported by a recent large-scale prospective cohort study (EMTICS, *n* = 259 children, with a first-degree relative with CTD, but no tics at entry at the age of 3–10 years) [75]. A positive relationship seems clearer concerning infections in early childhood for ADHD. (3) Direct evidence of pro-inflammatory overactivity in the brain in TD and ADHD is modest and limited and (4) hyper-reactivity of systemic immune pathways and neuro-inflammation may contribute to natural fluctuations of the core behavioral features of TD and ADHD. However, data and a specific comment on TD + ADHD co-occurrence is missing, although Chen et al. [44] reported on a Taiwan National Health Insurance research database (*n* = 5811 ADHD-only, *n* = 1816 TD-only, *n* = 349 TD + ADHD) that patients with TD + ADHD had a significantly higher prevalence of allergic diseases than (in decreasing order) ADHD-only, TD-only, and controls, suggesting that ADHD is the essential factor.

Finally, the role of vitamin D (25(OH)D) serum level in comorbid TD + ADHD is unclear, although observational studies and a meta-analysis revealed a lower level in a clinically significant proportion (about 50%) of children with ADHD [76,77,78]. For clarification of transdiagnostic aspects, Bond et al. [79] investigated 25(OH)D in three groups (TD, *n* = 327; first degree relatives with tics, *n* = 31; first degree relatives without tics, *n* = 93). Lower levels of vitamin D were not associated with tics but only with the presence and severity of ADHD in children and adolescents with TD, pointing to separate effects of ADHD in TD + ADHD, supporting independence of the two factors. 

Moreover, vitamin D deficiency might modify dopaminergic pathways and thus influence ADHD symptoms [80]. Whether or not vitamin D supplementation is of any clinical help needs further studies [78,81]. On the other hand, the physiological effects of vitamin D on calcium homeostasis/bone metabolism are established and, probably, there exists an inhibitory role of vitamin D in the regulation of erythropoesis and systolic blood pressure in adolescents with ADHD [80], which is a possible line of “psycho-somatic” developmental research to be followed.

### 3.3. Neurophysiology

Patients with TD, ADHD, and TD + ADHD have problems with controlling their movements in line with the hypothesis of impaired motor inhibition in TD, ADHD, and TD + ADHD [82]. Pure neuropsychological studies with, for example, Flanker or stop tasks (e.g., [83,84,85] only allow indirect conclusions about the underlying neuronal processes in contrast to using neurophysiological concepts such as EEG oscillations and event-related potentials (ERPs), transcranial magnetic stimulation (TMS) paradigms, or fMRI connectivity measured either at rest or during the execution of a task.

When Rothenberger and Heinrich [86] reviewed electrophysiological studies in children and adolescents with TD in order to describe their brain dynamics related to neuronal inhibition and facilitation, they usually found additive motor and cognitive effects for TD + ADHD, but they also considered other peculiarities of TD + ADHD:

In general, all the electrophysiological reports underline the good abilities of children with TD for neuronal/behavioral compensation of their motor hyper-excitability. The question arises as to whether this still holds when TD is associated with ADHD, because for ADHD-only inhibitory abilities are reduced (e.g., decreased theta waves and delayed event-related potential (ERP) latencies during tasks involving inhibitory abilities [87,88], reduced readiness potential [89], reduced contingent negative variation [90,91]). Fortunately, recent evidence of sufficient compensatory abilities in children with TD + ADHD comes from a neurofeedback study by Gevensleben et al. [65], where both tics and ADHD symptoms could be improved, and (concerning sufficient inhibitory control) from an electrophysiological EEG/ERP investigation by Morand-Beaulieu et al. [88] including four groups of children aged 7–14 years (TS-only, *n* = 47; TS + ADHD, *n* = 32; ADHD-only, *n* = 22; matched typically developing controls, *n* = 35).

This is in line with the experimental approach of Eichele et al. [92,93], who reasoned that a typical decline in tic severity as well as an increase in ability to suppress tics in late childhood and adolescence develop in parallel with general improvement of self-regulatory abilities and performance monitoring. The authors used ERPs in a modified flanker task in three groups of children: TD (including TD + ADHD and TD without ADHD), ADHD, and TDC, performing two testing sessions administered, on average, 4.5 years apart [93]. All groups improved with task performance and ERPs (e.g., P300) over time, but only the TD group trajectories converged to normal with maturation, which is consistent with compensatory self-regulation mechanisms in TD. In a separate analyses, TD subgroups (TD + ADHD vs. TD without ADHD) were compared, but because of the small sample size (*n* = 7–14) the statistical power was low and differentiating factor-related results could not be reported.

Further, electrophysiological studies in the domain of cognitive control [18,94,95,96] were conducted in auditory sensory information processing with event-related potential indicators such as mismatch negativity (MMN), negative wave (Nd), P300, event-related theta oscillations (ERT), slow negative potential (SNP), and post-imperative-negative variation (PINV). All these studies, each between *n* = 44 to *n* = 53 children, included four groups (TD, ADHD, TD + ADHD, and typically developing probands). The authors revealed that, first, neurodynamic sufficiency in ADHD and TD + ADHD seems to be similarly impaired with ADHD as the responsible factor and, second, that if very high cognitive performance is requested, the two associated disorders seem to interact at the level of electrophysiology, while at lower levels of cognitive demand the two factors (i.e., TD vs. ADHD) may be separately and additively at work.

Other neurophysiological studies in children with a 2 × 2 factorial design (using event-related potentials or a cortical silent period of transcranial magnetic stimulation) also presented informative examples for the additivity of TD + ADHD, usually with the weakest results for ADHD and the comorbid group with TD + ADHD [52,53,97]. In a recent study, Rawji et al. [98] investigated healthy adults (*n* = 15) and 19 patients with primary tic disorders with a stop-signal-task and TMS applied to the motor cortex. They found that automatic but not volitional inhibition (proactive and reactive) was impaired in tic disorders. Corticospinal excitability while preparing for movement execution was not different in patients with tic disorders, indicating that they do not have an abnormally excitable motor output. Although comorbidity with OCD appeared to affect results, sample size (comorbid ADHD, *n* = 6; OCD, *n* = 7; OCD + ADHD, *n* = 3) was too small to draw firm conclusions.

The brain’s functional organization during resting state or task processing can also be captured by functional magnetic resonance imaging (fMRI), with the advantage of referring the results topologically to certain cortical and subcortical brain regions.

Openneer et al. [54] registered and compared, with a 2 × 2 factorial design and a whole-brain-fMRI approach, the data of *n* = 109, 8–12 year old children with TD (*n* = 27), TD + ADHD (*n* = 19), ADHD (*n* = 23), and HC (*n* = 40). Local efficiency and clustering coefficient were significantly lower in children with TD in the default mode network compared with HC, and in the fronto-parietal network compared with ADHD. The findings for TD + ADHD were more similar to TD than to HC, and thus may represent a TD-related deficit. This gives support for a lower short-range network connectivity in the default mode network of TD, assuming the misguided synchronization of neuronal oscillations, as already outlined by Rothenberger [99].

Another publication of this working group [100] evaluated 103 of these children (TD, *n* = 28; TD + ADHD, *n* = 23; HC, *n* = 52) with fMRI during a stop-signal task. They observed an impaired response inhibition performance in the group with TD + ADHD, while showing that ADHD severity was the driving force and neural activation during failed inhibition was stronger in TD + ADHD at the inferior frontal gyrus and temporal and parietal areas. Because a group with ADHD-only was missing, a 2 × 2 factorial design could not be conducted. Nevertheless, these data may help to topographically localize the networks of known inhibitory deficits in TD + ADHD and ADHD (see [21,101]).

A third paper of this fMRI-group from the Netherlands [55] investigated neural reward processing in 124 of these children (TD, *n* = 18; TD + ADHD, *n* = 29; ADHD, *n* = 29; HC, *n* = 48). Neural activation during reward anticipation and receipt was not associated with TD but with ADHD and is in line with the view of nucleus accumbens hypoactivation, related to transdiagnostic ADHD symptom severity and an additive model of TD + ADHD.

Polysomnographic sleep-laboratory registrations in children of the four groups in question (i.e., TD, ADHD, TD + ADHD, HC) confirmed the earlier findings of short arousals and tics during sleep in TD. When ADHD symptoms accompanied TD, in addition, nightly general motor restlessness and disorganized behavior may take place (e.g. Stephens et al. [58], with *n* = 90 children). Kirov et al. (with *n* = 18 children in each of the four groups) concluded that in children with TD + ADHD, sleep problems seem to be higher, having both REM sleep increase (for ADHD) and lower sleep efficiency/elevated arousal (for TD) in an additive manner [56,102]. More recently, Keenan et al. [57] conducted a systematic review and meta-analysis of polysomnographic findings on overlapping sleep disturbances in TD and ADHD, merging child and adult populations. Twenty studies met final inclusion criteria, combining TD (*n* = 108), ADHD (*n* = 316), TD + ADHD (*n* = 79), and HC (*n* = 336). Compared to HC, TD and TD + ADHD groups had significantly lower sleep efficiency and higher sleep onset latency. TD + ADHD also had significantly increased time in bed and total sleep time. No significant differences were observed between the highly heterogeneous ADHD group and HC. Hence, TD + ADHD was associated with more pronounced differences. Unfortunately, most of the studies evaluated in this meta-analysis did not include all the four groups and thus it was not possible to create a 2 × 2 design in order to determine which factor (i.e., TD vs. ADHD) was responsible for the results, although adding ADHD to TD might have led to the pronounced differences, as also seen in a wrist-worn, seven days accelerometer study on physical activity, sleep, and neuropsychiatric symptom severity in *n* = 110 children with TD with/without different psychopathologies including ADHD [103]. In this sense, Keenan et al. [57] suggest that “different sleep parameters impacted to varying degrees in each population”, which goes well with an additive model for TD + ADHD.

## 4. Conclusions and Perspectives

Only a few empirical studies on the neurobiological background of TD and ADHD have taken the peculiarity of co-existing TD + ADHD into consideration, and still fewer have used the methodological approach of a 2 × 2 factorial design in order to disentangle the impact/effects of TD vs. ADHD within this comorbidity.

All the neurobiological studies with a 2 × 2 design were conducted in children and adolescents. Therefore, the questioning of the long-term stability of the behavioral additive model in adults with earlier TD + ADHD by Müller et al. [19] needs to be confirmed before valid conclusions can be drawn.

However, some new studies in youngsters with a 2 × 2 design (and other studies including a TD + ADHD group) are available. Although not every problem is solved, existing data extend and strengthen the assumption that TD + ADHD may be considered basically as an additive model at the levels investigated (i.e., psychopathology, neuropsychology, genetics, neuroanatomy, neurochemistry, and neurophysiology). However, researchers are well aware that, while applying a clinical-categorical approach to this modelling, the ADHD core symptoms and associated psychopathology (e.g., emotional problems) are dimensional with certain clinical cut-offs but also sometimes showing subclinical/subcategorial values higher than in typically developing children. In addition, for example, attentional problems may hinder a child from suppressing his/her tics, or increased general motor restlessness might interfere with tics. Hence, some behavioral interdependence between the factors TD and ADHD have clinically to be taken into consideration but, overall, disorder-specific contributions to impairments shall guide targeted interventions for TD and ADHD within TD + ADHD [5].

Thus, for practical reasons, this favors a more dimensional approach to child psychiatric disorders, finally leading to a multilevel/multisymptomatic diagnostic-profiling as a helpful precondition for decisions on counselling and specific treatment programs within the framework of “personalized medicine”; in the case of TD + ADHD, a treatment “in parallel” at different levels such as psychoeducation, counselling, psychotherapy, and psychopharmacotherapy.

At the same time, the presented findings make clear that some overlapping (“non-additive”) deviations between TD and ADHD exist, which are of a more general neurobiological character, as can be seen for other early-onset neurodevelopmental disorders such as autism spectrum disorder. The parameters of these non-specific cross-disorder functional systems (such as stress system, immunologic system, endocrinologic system, regulation of blood pressure) may or may not be quantitatively different between TD, ADHD, and TD + ADHD (and sometimes modulate specific clinical core symptoms), while disorder-specific disturbances in certain subcortical–cortical neuronal circuits may lead to disorder-related symptoms and can finally induce TD + ADHD in a more or less additive way, including interactive effects when higher cognitive functions are demanded, i.e., TD + ADHD represents a clinical picture appearing higher than chance, expressing as a special co-occurring psychopathology without representing a separate diagnostic entity. Such reasoning should be studied and discussed further, helping to develop a positive perspective for a fruitful joint neurobiological and clinical research on TD + ADHD. In this respect, a large-scale European Multicenter Tics in Children Study (EMTICS) that is currently underway in order to explore different aspects of TD (e.g., precursors of tics, clinical presentation in males vs. females) will probably shed further light on the associations of TD + ADHD [104,105].

## Figures and Tables

**Table 1:** Independence of deviations for biological parameters and environmental factors within the framework of an additive model of TD + ADHD; selectively for TD + ADHD or one of its two factors.

Level	TD + ADHD	TD	ADHD	Reference
“Genetical & environmental risk factors”				
Genetics		*11q24* *KCNJ5*	*11q24* *KCNJ5*	Gomez et al. [32]
		13 genome wide significant regions	13 genome wide significant regions	Yang et al. [35]
	*ADRA2A* genotypic distribution allelic transmission			Xu et al. [39]
Environment	Early risk factors/pre- and perinatal events (mainly related to the factor ADHD)	Early risk factors/pre- and perinatal events (subclinical ADHD?)	Early risk factors/pre- and perinatal events	Hoekstra et al. [40] Brander et al. [42] Chen et al. [44]
“Abnormal brain conditions”				
Brain structure		caudate nucleus ↓ interhemispheric connectivity ↓		Plessen et al. [45]
			Amygdala ↓?	Ludolph et al. [46] Wittforth et al. [47]
			Grey matter volume ↓	Draganski et al. [48]
Neurochemistry		TIQs (↑ ?)		Capetian et al. [49]
		D1-receptor agonist active		Gilbert et al. [50]
		Endocannabinoids ↑		Müller-Vahl et al. [51]
Neurophysiology	Early event-related theta oscillations ↑	CSP ↓	ERP components (e.g, P3) ↓, Theta- oscillations ↑, ICF/ICI ?	Yordanova et al. [18] Moll et al. [52] Shepard et al. [53]
		Lower short-range network connectivity		Openeer et al. [54]
			Nucleus accumbens hypoactivation	Akkermans et al. [55]
		Sleep: arousal ↑, efficiency ↓ tics ↓	REM-sleep ↑? General movements ↑	Kirov et al. [56] Keenan et al. [57] Stephens et al. [58]

Legend: ↓ decreased, ↑ increased, ? questionable, KCNJ5 Potassium Inwardly Rectifying Channel J5, *ADRA2A* Alpha-2A adrenergic receptor, TIQs Tetraisochinoline, ERP Event-Related Potential, CSP Cortical Silent Period, ICI/ICF Intracortical Inhibition/Facilitation, REM Rapid Eye Movements.

## Data Availability

Not applicable.
